# Spermatozoa Observed a Second Time within the Ovum

**Published:** 1843-06

**Authors:** Martin Barry


*Spermatozoa Observed a Second Time within the Ovum.*—[By Martin Barry, M.D., F.R,SS. L. & E.]—Several months since I communicated to the Royal Society the fact that I had observed, and shown to Professor Owen and others, spermatozoa *within* the mammiferous ovum. The ova were those of the rabbit, taken twenty-four hours *post coitum* from the Fallopian tube.

I have this day (March 31) confirmed the observation; several ova from the Fallopian tube of another of these animals, in a somewhat earlier stage, having presented spermatozoa in their interior, *i. e.* (as the first observation) within the thick transparent membrane ("*zona pellucida*") brought with the ovum from the ovary.—*London Lancet.*

